# Identifying Predictors of Gambling Episodes and Craving Using Ecological Momentary Assessment and Smartwatch-Based Physiological Measures: Protocol for a Longitudinal Observational Study

**DOI:** 10.2196/82782

**Published:** 2026-02-19

**Authors:** Andreas Maximilian Meyer, Esther Kim, Hae Kook Lee, Gwanghyun Jo, Michael Patrick Schaub, Severin Haug

**Affiliations:** 1 Swiss Research Institute for Public Health and Addiction (ISGF), University of Zürich, Konradstrasse 32, Zürich, 8005, Switzerland, 41 444481180; 2 Department of Counseling Psychology, Korea Baptist Theological University, Daejeon, Republic of Korea; 3 Department of Psychiatry, Uijeongbu St. Mary’s Hospital, The Catholic University of Korea, Seoul, Republic of Korea; 4 Department of Mathematical Data Science, Hanyang University ERICA, Ansan-si, Republic of Korea

**Keywords:** gambling, craving, biometric monitoring technologies, ecological momentary assessment, ecological momentary intervention, just-in-time adaptive intervention, wearable, smartwatch, prediction, BioMeTs, EMA, EMI, JITAI

## Abstract

**Background:**

Like other addictive behaviors, problem gambling is often chronic and relapsing. While digital interventions offer low-threshold treatment and support, their effectiveness is often limited by small effect sizes, low adherence, and high dropout rates. Progress in digital technology has enabled the development of ecological momentary interventions (EMIs), which provide just-in-time support tailored to users’ needs. However, EMIs for addictive behaviors have hardly been developed so far.

**Objective:**

This study aimed to explore and identify relevant predictors of gambling episodes assessed by ecological momentary assessments and physiological smartwatch (Apple Watch) data, which in turn may be used for the further development of EMI-based interventions.

**Methods:**

A total of 109 at-risk gamblers were recruited online in a collaborative study between Switzerland and Korea. Over a period of 28 days, participants were asked to complete brief ecological momentary assessment surveys 3 times a day (morning, afternoon, and evening) asking about their gambling behavior and their levels of craving intensity, sleep quality, physical activity, boredom, vitality, depression, and anxiety. They were instructed to wear an Apple Watch that continuously and passively recorded several physiological indicators (eg, heart rate [variability], sleep metrics, and physical activity). Machine learning techniques and multilevel modeling approaches will be used to develop prediction models for gambling episodes and to identify relevant predictors.

**Results:**

Data collection has been completed since April 2025. In total, 109 participants have been enrolled in both countries (52 in Switzerland, 57 in Korea), and datasets are currently being prepared for analysis. The collected data are expected to enable the development of prediction models for gambling episodes.

**Conclusions:**

Incorporating the relevant predictors found in this study into digital intervention programs and providing just-in-time individually tailored intervention elements could improve program engagement and effectiveness. The approach used in this study is transferable to other digital interventions for addictive behaviors and holds promise to exploit their potential.

## Introduction

Mental disorders and addictive behavior contribute significantly to the global burden of disease [1]. Although depressive disorders and the use of addictive substances continue to be highly prevalent, behavioral addictions are becoming increasingly relevant. Particularly, the availability, participation, and cost of gambling have increased dramatically in recent years, with smartphones being used more and more to access online gambling [2]. Gambling is considered a form of entertainment characterized by betting mechanisms and monetization aspects, and habitual gambling activity has the potential to cause severe distress or impairment [3,4]. The first gambling research that incorporated burden of disease approaches concluded that the harm associated with gambling was comparable to the harms due to depression and alcohol use disorders and even greater than the harm associated with drug dependency and a number of widespread chronic physical conditions [5]. On the individual level, problem gambling is linked to impaired work or academic performance, social deviance, marital problems, emotional and psychological distress, and a variety of mental health conditions, such as personality disorders, alcoholism, anxiety, and mood disorders [6]. However, the harm negatively impacts not only the gamblers themselves but also their families and the community [7,8]. As problem gambling is associated with debt, loss of productivity, and crime, and disrupts family functioning by eroding the time and attention given to the partner, children, and family responsibilities, a person’s gambling harms multiple significant other persons, adding to the total burden of gambling harm in the population [9,10]. Gambling disorder was recently included as a new condition in the *ICD-11* (*International Classification of Diseases, 11th Revision*). Particularly economically and socially disadvantaged populations experience gambling disorder and harms more frequently; therefore, problem gambling is likely to aggravate other health and socioeconomic imbalances and disparities [11].

According to estimates for 2016, the prevalence of problem gambling ranged from 0.2% to 5.3% across various nations [3]. Based on the latest representative data from 2022 and diagnostic criteria of the *DSM-5* (*Diagnostic and Statistical Manual of Mental Disorders* [Fifth Edition]), the estimated lifetime prevalence of at-risk gambling in Switzerland is 5.8%, and the prevalence of pathological gambling is 0.8%. In relation to the past 12 months, the estimated prevalence for at-risk gambling is 3.8% and for pathological gambling, 0.5% [12].

Similar to other addictive behaviors, problem gambling is typically chronic and relapsing, with evidence to suggest that over half of all incident problem-gambling cases are previous problem gamblers who are relapsing [13]. Evidence for gambling interventions that can lower opportunities for potentially hazardous gambling has been found; however, there’s a lack of screening and brief interventions providing ongoing support to reduce gambling-related harm and relapse [14].

Although face-to-face psychosocial interventions for the treatment of gambling problems show small but statistically significant reductions in gambling behavior [15], only a minority of affected individuals seek professional treatment services, with fewer than 10% of problem gamblers seeking professional help, as obtained in a German study [16]. Potential barriers to obtaining professional help include the lack of services, the stigma attached to addictive behavior, sociocultural obstacles, privacy concerns, and a preference for self-reliance [17]. In comparison to face-to-face treatment, digital interventions have a variety of potential advantages, including ready accessibility, perceived anonymity, individually tailored contents, and low costs. Recent systematic reviews suggest that internet-based treatment has the potential to reach a large proportion of persons with gambling problems and that this approach shows promising effects on general gambling symptoms, gambling frequency, and amount of money lost gambling [18,19]. The majority of the available internet-based interventions rely on principles derived from cognitive behavioral therapy and motivational interviewing and typically include monitoring of gambling behavior, goal setting as well as information on protective behavioral strategies to cope with stress and cravings [18-20]. Although current internet-based interventions are an effective low-threshold treatment option for problem gamblers, their effect sizes are usually small and program adherence is low, including low program use and a high dropout rate. One potential reason for the limited effectiveness and engagement of current digital interventions for problem gambling is their provision of primarily static content and their lack of dynamic tailoring that aims to provide the right type and amount of support to people in their everyday lives [18,19].

In view of this, there is an urgent need for innovative digital approaches that respond more dynamically to the individual needs of those affected and offer support precisely when it is needed most. One such approach—ecological momentary interventions (EMIs)—delivers behavior change support just in time when users most want or need the support. The addition of EMI components to existing digital interventions for the treatment of addictive behaviors holds the promise of increasing their use and efficacy because of the capacity to track the interaction between one’s internal states, such as feelings and cravings and the addictive behavior (eg, a gambling episode). The relapse prevention model, a prominent social-cognitive theory developed to explain relapse in substance use disorders, categorizes elements or circumstances that may trigger or aid relapse or addictive behaviors [21]. This model’s major premise is that high-risk situations, such as unfavorable emotional states, interpersonal conflict, social pressure, and cravings, precede lapse and relapse. Recent empirical studies indicate that craving for gambling is a multifaceted construct that is characterized by mental imagery, desire thoughts, and physiological sensations and triggered by a variety of stimuli, including positive affect, negative affect, external cues, mental imagery, and desire thoughts [22]. A recent Australian ecological momentary assessment (EMA) study revealed that gambling cravings predicted gambling episodes and craving self-efficacy predicted gambling expenditure [23]. Based on these findings, Hawker et al [24] recently developed a smartphone-delivered EMI, including EMAs comprising 10 self-report items, assessing major constructs of the relapse prevention model 3 times daily. Although results of this pre-post pilot study showed some beneficial intervention effects, the compliance rates for the EMA (51%) and EMI (15%) were low [24]. This result is in line with other studies indicating high attrition and low retention rates in app-delivered interventions for mental health [25].

Although EMIs hold significant therapeutic promise, low user compliance remains an obstacle. To address this issue and simultaneously ensure continuous monitoring of relevant parameters, passive measurement methods, such as biometric monitoring technologies (BioMeTs), are becoming increasingly important as they require less active involvement from users and thus provide a promising alternative to self-reports for EMA studies and EMIs. According to Goldsack et al [26], BioMeTs are “connected digital medicine products that process data captured by mobile sensors using algorithms to generate measures of behavioral and/or physiological function.” Advantages over self-reports include the potential of BioMeTs to be less burdensome (due to passive data collection), less challenging to complete (due to cognitive or disease-specific impairments), less focused on perceived emotional states (given that they rely on physiological or behavioral measurements), less location-reliant (ie, remote assessment is possible), and less stigmatized (eg, when using a clinical assessment). The challenges include that the monitoring devices (eg, wearables, such as wristbands or smartwatches) have to be worn for a longer period and that the physiological and behavioral indicators collected are still poorly validated in some cases [26]. However, there has been tremendous progress recently both in the validation of valid physiological and behavioral indicators and in the availability of low-cost devices, like wristbands, that can collect these indicators with high reliability. The use of wearable devices like wristbands or smartwatches to track mood and behavior brings enormous potential for EMIs. They provide continuous data on behaviors essential to psychological and health assessments, such as sleep and wake cycles, activity, movement, and stress by passively monitoring motion, heart rate, and other physiological factors [27].

At present, this field of research is in the process of development and there are several studies showing associations between depression and digital features from sleep, physical activity, location, and phone use data [27]. However, there are only a few studies in the field of addictive behavior and so far no studies in the field of gambling, although the passive collection of stress via wearables could be particularly relevant. Gambling and gambling cravings are associated with stress in various ways: On the one hand, gambling behavior can serve as a distraction from and a means to cope with stress; on the other hand, gambling-related losses and harm can become a source of stress, which may lead to a feedback cycle leading to more gambling and more stress [28]. Many studies underline the close association between stress and gambling, which is also expressed in the diagnostic criteria for gambling disorder (ie, “gambling when feeling distressed,” which is a *DSM-5* criterion for gambling) or a study among gamblers showing that self-reported stress predicted gambling craving over and above reports of anxiety or depression [29].

This study was designed as an international collaboration between the research teams of the Swiss Research Institute for Public Health and Addiction (Switzerland), Uijeongbu St. Mary’s Hospital of the Catholic University, and Korea Baptist Theological University (both in Korea) and aims to identify potential physiological and psychological indicators that can reliably predict gambling episodes and cravings, using data collected via self-reported EMA surveys and an Apple Watch. Unlike previous studies focusing mainly on self-reported data, this study additionally leverages the potential of BioMeTs, such as passively collected physiological data from an Apple Watch (eg, heart rate [variability], sleep metrics, and physical activity), to examine their role as predictors of gambling-related outcomes.

## Methods

### Objectives

This study aims to explore and identify relevant predictors of gambling episodes in a general population sample of at-risk gamblers by:

1. Collecting physiological and psychological indicators (levels of craving intensity, sleep quality, physical activity, stress, vitality, boredom, depression, and anxiety) 3 times a day using brief online EMA self-reports
2. Collecting physiological indicators (eg, heart rate [variability], sleep metrics, and physical activity) passively and continuously throughout the day using a 2024 2nd generation Apple Watch SE (Special Edition)
3. Developing prediction models for gambling episodes using machine learning (ML) algorithm technique, including time series forecasting (TSF) and recurrent neural networks (RNNs), as well as multilevel modeling approaches, such as generalized linear mixed-effects models (GLMMs) and multilevel vector autoregression (mlVAR) models, based on the collected indicators.

### Study Design and Procedure

**Figure 1.**
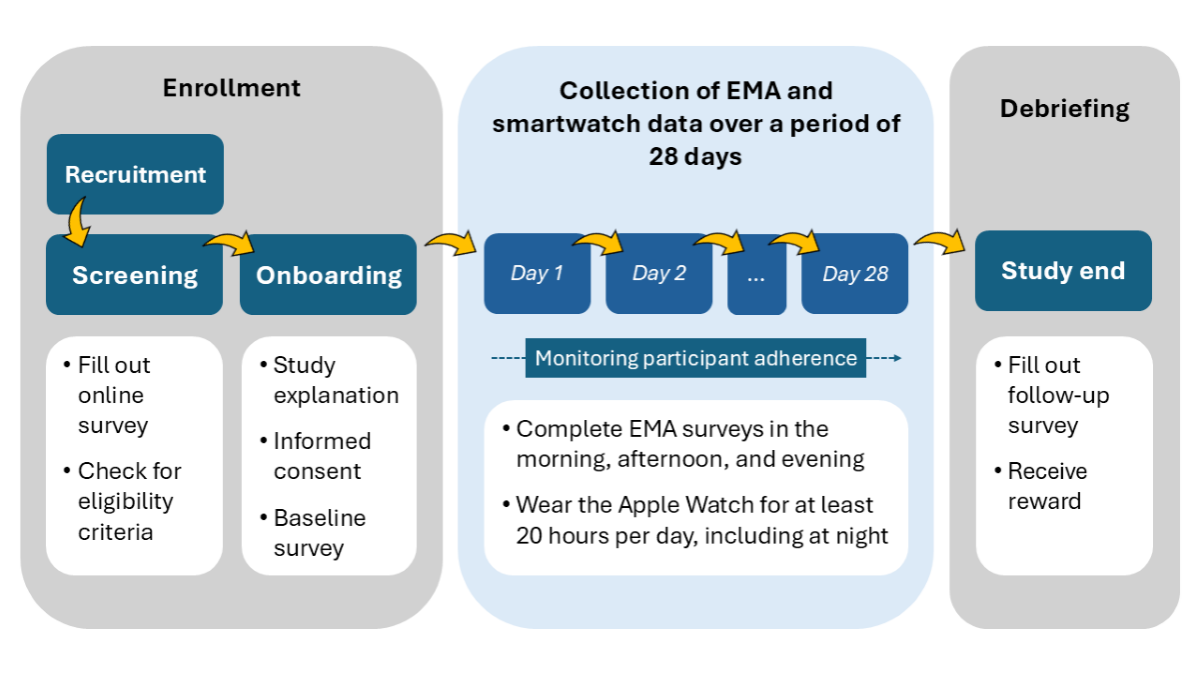
Study procedure. EMA: ecological momentary assessment.

This longitudinal observational prediction study combined prospective observational and noncontact research methods. Self-reported EMA surveys were conducted over a period of 28 days (4 weeks) using individuals’ mobile phones to assess gambling behavior and craving-related internal states as well as physiological and psychological variables (emotions, physical activity, stress, vitality, and sleep status) 3 times a day (morning, afternoon, and evening). Over the same period, physiological variables (eg, activity, heart rate, and sleep status) were continuously and passively measured using a 2nd generation Apple Watch SE, including at night to record sleep data. The study procedure is outlined in Figure 1, which provides an overview of the entire process, including the enrollment of participants, the collection of EMA and physiological smartwatch data, and the debriefing at the end of the study.

#### Recruitment and Screening of Study Participants

Between February and April 2025, a total of 52 Swiss participants (mean age 37.4, SD 11.9; range 18‐79 years; female: n=5, 9.6%) were recruited via a customer satisfaction survey conducted by Swiss Casinos AG that was sent to all online casino guests residing in the German-speaking part of Switzerland. In Korea, a total of 57 participants (mean age 25.6, SD 4.5; range 19‐33 years; female: n=29, 50.9%) were recruited between October and December 2024 through a professional research agency via online advertisements. Thus, a total number of 109 participants were recruited in both countries, meeting the requirement of reaching the target sample size of 100 participants (refer to “Sample Size” in the “Methods” section for further details regarding the sample size). In both countries, candidates who wished to participate in the study were informed of its aim, and those who consented were screened online for eligibility. The inclusion criteria were (1) age ≥18, (2) a Problem Gambling Severity Index (PGSI) score ≥3, (3) gambling at least once a week, and (4) cognitive capacity to reply to short online EMA surveys and use an Apple Watch. Exclusion criteria were (1) significant suicide risk according to the P4 suicidality screener [30] and (2) current psychiatric illness requiring psychiatric medication or outpatient treatment more than twice a month or current hospitalization in a psychiatric unit.

#### Onboarding

In both countries, interested participants were screened for eligibility via an online survey to determine if they met the inclusion criteria. Individuals who met the eligibility criteria and expressed interest in participating received detailed information about the purpose of the study, procedures, potential risks and benefits, and participant rights. Those who agreed to participate were invited to a personal onboarding appointment, which was conducted in groups. At the appointment, they were informed of the study, filled out the informed consent form, completed the baseline assessment, and received an Apple Watch. The baseline assessment was a web survey that measured sociodemographic information (eg, age and gender) and problem gambling-related indicators, such as participants’ level of problem gambling, gambling symptom severity, frequency of each type of gambling, platform used for gambling (online and offline), and revenue (total profit or loss made gambling). They were also asked about physiological indicators, such as sleep quality, physical activity, and stress, as well as psychological indicators, such as depression and anxiety. Co-occurring symptoms and conditions, such as alcohol use, substance use, and attention deficit and hyperactivity, were also measured (refer to “Baseline and Follow-Up Assessments” in the “Measurements” section for an overview and further details regarding the baseline instruments used in this study). After completing the baseline survey, participants were asked to complete brief EMA self-report surveys, which they received on their smartphones 3 times a day, and wear an Apple Watch for at least 20 hours a day, including at night while sleeping, for 28 days.

#### Collection of EMA and Smartwatch Data

**Figure 2.**
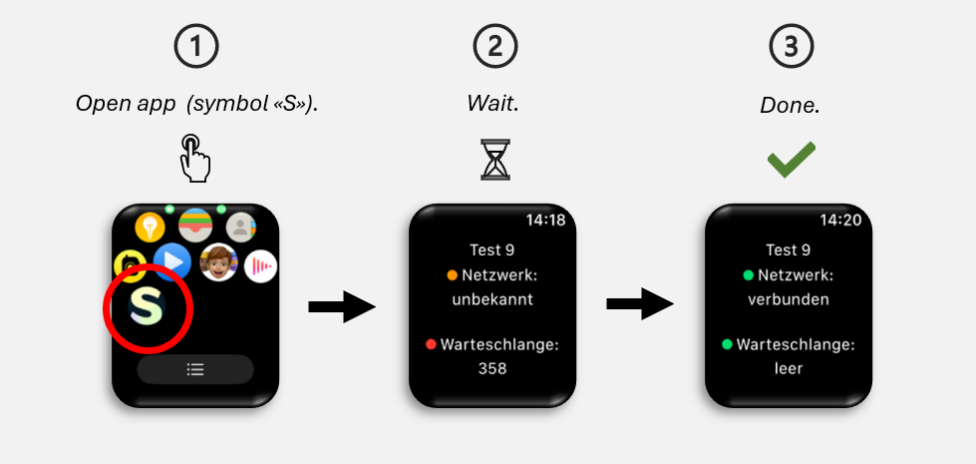
Customized stand-alone app and its data synchronization steps from the Swiss participants’ perspective.

To capture real-time data about participants’ internal states, they received links to brief self-report EMA surveys on their mobile phones three times a day: (1) in the morning from 6 AM to noon, (2) in the afternoon from 3 PM to 5 PM, and (3) in the evening from 8 PM to 10 PM, over a period of 28 days. In case of nonresponding, reminders were sent out at 7:30 AM, 9 AM, and 10:30 AM (for the morning EMA), at 4 PM (for the afternoon EMA), and at 9 PM (for the evening EMA). Participants were asked about their gambling behavior, such as whether they had gambled the previous day or were currently gambling. They were also asked about their craving intensity, physical activity, sleep quality, current stress levels, vitality and boredom levels, as well as their anxiety and depression levels (refer to “Self-Reported EMA Assessments” in the “Measurements” section for an overview and further details regarding the EMA assessments used in this study). In addition, participants were instructed to wear an Apple Watch for at least 20 hours a day. The device continuously and passively collected their physiological data, such as heart rate, heart rate variability, and sleep metrics, over a period of 28 days (refer to “Measurement of Physiological Indicators” in the “Measurements” section for an overview and further details regarding the physiological indicators collected by the Apple Watch). A 2024 2nd generation Apple Watch SE was used to collect participants’ physiological data. It was emphasized that the smartwatch should be worn at night while sleeping in order to record sleep data, such as sleep patterns and cycles. In Switzerland, participants collected relevant physiological data on their Apple Watch using a customized stand-alone app that was specifically designed for the purpose of this study. Swiss participants were required to actively synchronize the data measured and collected throughout the day by the Apple Watch using the study app’s synchronization feature once per day (refer to Figure 2 for the data synchronization steps from the participants’ perspective). In Korea, participants used a mobile app developed for the collection of both EMA and physiological data. Data from the participants’ Apple Watches were accumulated through the mobile app, which was integrated with Apple’s Health app. When participants received an EMA notification and accessed the app to complete the brief survey, this action was designed to automatically synchronize the data from the watch.

#### Monitoring Participant Adherence and Compliance

In Switzerland, individuals’ adherence to study participation (ie, completing as many of the EMA self-report surveys as possible each day and wearing the Apple Watch for at least 20 hours per day, including at night to record sleep data) was actively monitored. Participants with low adherence were encouraged to complete the EMA self-report surveys and wear the Apple Watch via notifications or proactive phone calls. In Korea, the mobile app was designed to automatically send reminder notifications if a participant failed to complete an EMA survey. Physiological data were also synchronized daily, enabling the research team to monitor if the previous day’s data had been collected. For participants who did not meet these collection criteria, a reminder phone call was made to encourage them to wear the Apple Watch.

#### Debriefing and Follow-Up Assessments

Participants received the follow-up questionnaire after the study period of 28 days had ended (ie, on the 29th day) and used the same measures as in the baseline assessment, along with an additional questionnaire designed to assess whether participants felt influenced by the study itself—for example, whether wearing the Apple Watch affected their sleep or whether the frequent reminders about gambling led to increased gambling behavior (refer to “Baseline and Follow-Up Assessments” in the “Measurements” section for an overview and further details regarding the follow-up instruments used in this study). Participants were then able to choose their participation reward. For active participation, Swiss participants received either a pure monetary reward of up to CHF 400 (US $515) or the Apple Watch, including a pro rata amount of money (the purchase price of the Apple Watch was deducted from the total reward amount), whereas Korean participants received a KRW 100,000 (US $68) cash reward and the study’s Apple Watch. The total amount of the reward depended on the level of engagement in the study (eg, the number of daily EMA surveys completed or the number of times participants wore the Apple Watch for at least 20 h/d). In both countries, compensation was determined by compliance, with distinct dropout criteria for EMA and Apple Watch usage. Participants were dropped without compensation if nonadherence—either completing fewer than 3 daily EMAs or wearing the Apple Watch for less than 20 hours—occurred for 2 or more consecutive days or on 3 or more occasions within a week. These 2 criteria were assessed independently. Participants who avoided dropout and maintained an overall compliance rate of 70% or more.

### Hypotheses

Study hypotheses were formulated with respect to the main outcome of interest (ie, whether participants gambled the day before).

1. Psychological indicators (depression, anxiety, and stress) are associated with certain patterns of gambling episodes and cravings, and this information can be used to predict gambling behavior.
2. The physiological indicators (stress, sleep duration and quality, vitality, and physical activity) are associated with certain patterns of gambling episodes and craving, and this information can be used to predict gambling behavior.
3. Physiological indicators continuously measured with the Apple Watch are more predictive of gambling episodes than those measured by a self-reported online EMA survey.

### Measurements

Measurements of this study included baseline and follow-up assessments before and after the study, brief self-reported EMA surveys 3 times daily, and continuous recording of the physiological smartwatch data at least 20 hours a day over a period of 28 days. Data were collected independently in both countries using standardized and harmonized procedures and assessment instruments (eg, items of baseline, follow-up, and EMA surveys), allowing for cross-cultural comparison.

#### Self-Reported EMA Assessments

##### Overview

Below, the specific EMA items and their respective characteristics are described (refer to Table 1 for an overview of the EMA items and their schedule).

**Table 1.** Items for EMA^a^ and dissemination schedule.

Item	Time point			Answer options
	Morning (6 AM-noon)	Afternoon (3 PM-5 PM)	Evening (8 PM-10 PM)	
Gambling episode^b^	✓			Dichotomous (yes or no)
Currently gambling	✓	✓	✓	Dichotomous (yes or no)
Craving intensity	✓	✓	✓	Not at all (0)‐very severe (5)
Sleep quality^b^	✓			Terrible (0)‐excellent (10)
Physical activity^b^	✓			Dichotomous (yes or no)
Stress level	✓	✓	✓	Not at all (0)‐very strongly (5)
Vitality level	✓	✓	✓	Not at all (0)‐very strongly (5)
Boredom level	✓	✓	✓	Not at all (0)‐very strongly (5)
Depression level	✓	✓	✓	Not at all (0)‐very severely (5)
Anxiety level	✓	✓	✓	Not at all (0)‐very severely (5)

a EMA: ecological momentary assessment.

b Items “Gambling episode,” “Currently gambling,” “Sleep quality,” and “Physical activity” refer to the previous day and were therefore only asked once per day.

##### Gambling Episode

The primary outcome of interest, the gambling episode, was assessed during the morning EMA survey. Participants were asked: “Did you engage in gambling yesterday?,” a dichotomous variable with response options “Yes” and “No.” If participants chose “Yes,” they were asked follow-up questions about which platforms they used for gambling (online and offline), the type of gambling they engaged in (eg, casino games, such as blackjack), and the specific times at which they gambled.

##### Craving

Participants’ craving levels were measured 3 times daily (morning, afternoon, and evening) using the item “How strong is your urge to gamble right now?” Responses were recorded on a Likert scale ranging from 0 (“Not at all”) to 5 (“Very strong”). Craving was only measured if participants stated that they were not currently gambling at this very moment, using the item “Are you gambling right now?” (answer options: “Yes” or “No”).

##### Physical Activity

Physical activity was measured only during the morning EMA, asking participants “Yesterday, have you done a total of 15 min or more of physical activity, which was enough to raise your breathing rate.” Participants could choose from “Yes” or “No” as an answer. When choosing “Yes,” a follow-up question that asked participants about how many minutes (range 15-300) they had invested was presented.

##### Sleep quality

Sleep quality was also assessed only during the morning EMA, asking participants “Today, how would you rate your sleep quality over all (such as how many hours of sleep you got, how easily you fell asleep, how often you woke up during the night, how often you woke up earlier than usual)?” Answers ranged from 0 (“Terrible”) to 10 (“Excellent”).

##### Stress

The stress item measured participants’ stress level 3 times daily (morning, afternoon, and evening) using the following question: “Stress means a situation in which a person feels tense, restless, nervous, or anxious or is unable to sleep at night because one’s mind is troubled all the time. Do you feel this kind of stress right now?” Answers ranged from 0 (“Not at all”) to 5 (“Very strongly”).

##### Vitality and Boredom

Vitality and boredom levels will be measured 3 times daily (morning, afternoon, and evening). Questions include “How energetic and rested do you feel right now?” and “How bored are you feeling right now?” Answers ranged from 0 (“Not at all”) to 5 (“Very strongly”).

##### Depression and Anxiety

The psychological indicators, depression and anxiety, were measured 3 times daily (morning, afternoon, and evening). Questions include “Right now, how much are you bothered by feeling sad, down, or uninterested in life?” and “Right now, how much are you bothered by feeling anxious and nervous?” Answers ranged from 0 (“Not at all”) to 5 (“Very severely”).

### Measurement of Physiological Indicators

#### Overview

The physiological indicators measured by the Apple Watch and their specific characteristics are described below (refer to see Table 2 for an overview of the physiological indicators collected by the Apple Watch).

**Table 2.** Overview of physiological indicators measured by the smartwatch.

Category and indicator	Measurement frequency	Unit
Vital signs		
Heart rate	Continuously	bpm^b^
Heart rate variability	Sporadically (eg, during resting states)	ms
Sleep		
Time being awake	When sleep state is detected	min
Time being in bed	When sleep state is detected	min
Total time being asleep	When sleep state is detected	min
Core sleep duration	When sleep state is detected	min
Deep sleep duration	When sleep state is detected	min
REM^a^ sleep duration	When sleep state is detected	min
Activity		
Stand time	Continuously	min
Step count	Continuously	count
Active energy burned	Continuously	kcal
Distance by walking or running	Continuously	km
Physical activity (eg, workout)	During physical activity (eg, running)	min

a REM: rapid eye movement.

b bpm: beats per minute.

#### Vital Signs

Vital signs were recorded using the photoplethysmography sensor of the Apple Watch [31]. Participants’ heart rate (HR) was continuously and periodically recorded in beats per minute. When being inactive, HR measures intervals ranging from 5 to 10 minutes. During a workout or movement (eg, when the watch registers being in an active state), the measurement frequency increases to approximately 1-5 seconds. HR data will be used to assess participants’ physiological arousal and potential stress responses. The heart rate variability (HRV) was measured using the device’s optical sensors (photoplethysmography) to detect interbeat intervals between heartbeats (ie, the time intervals between heartbeats) over a certain period of time (approximately 60 seconds). HRV is calculated by determining the SD of the obtained interbeat intervals in milliseconds. The Apple Watch usually measures HRV during periods of rest or low activity (eg, while falling asleep, upon waking up, or during a breathing exercise). HRV will be used to gain insights into autonomic nervous system functioning and stress regulation.

#### Sleep Metrics

Sleep metrics were measured in minutes and collected automatically during sleep time using the device’s sleep tracking functionality [32]. To estimate sleep stages and sleep-related parameters, the device uses a combination of sensors, including an accelerometer, gyroscope, and HR monitor. The time being awake, the total amount of time a participant is awake during a sleep period, is recorded when the device registers transitions between sleep states and periods of wakefulness. The time being in bed is the total amount of time a participant spends in bed, including both wake and sleep periods. The time being asleep is the total amount of time spent asleep across all 3 sleep stages—core, deep, and REM sleep—all of which are based on HR and movement patterns during the night.

#### Activity Metrics

Activity metrics were automatically recorded using the Apple Watch SE’s built-in sensors, including the accelerometer, gyroscope, and HR monitor [31]. These metrics provide detailed insights into participants’ physical activity levels and patterns throughout the day. Stand time measures the amount of time (min) the participant has spent standing. Step count reflects the total number of steps taken by participants throughout the day. Active energy burned indicates the total amount of energy burned through physical activity throughout the day and is measured in kilocalories (kcal). This may include calories burnt from physical workouts or other nonsedentary activities. Both walking and running distances are measured continuously in kilometers (km) and provide information about participants’ nonsedentary activities (ie, the distance the user has moved by walking or running). Physical activities, such as running, cycling, or strength training, are actively tracked by the participant or detected automatically by the smartwatch based on movement patterns and HR. Duration (min), type of workout, and intensity were tracked for each recorded session.

### Baseline and Follow-Up Assessments

For the purpose of the study, reference periods of the baseline and follow-up instruments were standardized. All instruments were adapted to refer to the past 28 days, regardless of their original wording. Below, the instruments and their specific characteristics of the baseline and follow-up assessment are described (refer to Table 3 for an overview of the baseline and follow-up instruments used in this study and their schedule). In Korea, the respective Korean versions of the instruments were used.

**Table 3.** Screening, baseline, and follow-up instruments and their schedule.

Instrument	Screening	Baseline	Follow-up
Sociodemographics		✓	
Problem Gambling Severity Index (PGSI)^a^	✓	(✓)	✓
Gambling Symptom Assessment Scale (G-SAS)		✓	✓
Frequency by Gambling Type		✓	✓
Revenue (Total profit or loss)		✓	✓
Sleep quality		✓	✓
Physical activity (level of exercise)		✓	✓
Perceived Stress Scale (PSS-4)		✓	✓
Patient Health Questionnaire (PHQ-9)		✓	✓
General Anxiety Disorder (GAD-7)		✓	✓
Alcohol Use Disorder Identification Test (AUDIT-C)		✓	✓
Nicotine, Cannabis, and other drugs use		✓	✓
Adult Self-Report ADHD Scale (ASRS)		✓	✓
Suicidality Screener (P4-SCR)	✓		✓
Study Effects Questionnaire			✓

a The Problem Gambling Severity Index (PGSI) was used to screen for study eligibility, but also served as a baseline measure.

1. Sociodemographics included gender, age, country of birth (of mother, father, and participant), level of education, partnership status, and marital status.
2. The PGSI contains 9 items that assess a broad array of problems experienced by individuals who engage in problem gambling (eg, feelings of guilt, financial problems, and so on) [33,34]. The PGSI is the most widely used self-report measure of gambling harms in the literature. The total PGSI score was used as a screener in this study to capture participants’ level of problem gambling severity, which needed to be ≥3. Answers were given on a 4-point Likert scale ranging from 0 (“Never”) to 3 (“Always”). Scores range from 0 to 27 (0=nonproblem gambler, 1 to 2=low risk gambler, 3 to 7=moderate risk gambler, 8+=problem gambler).
3. The Gambling Symptom Assessment Scale (G-SAS) measured the severity of gambling symptoms, focusing on frequency, intensity, and duration of gambling urges, thoughts, behaviors, and both emotional and cognitive impacts [35,36]. Contains 12 items scored 0 (eg, “None”) to 4 (eg, “Extreme”). The total score ranges from 0 to 48 (higher scores=greater severity).
4. The Frequency by Gambling Type is a self-developed questionnaire designed to assess participants’ engagement in gambling activities, such as lotteries, casino games (eg, blackjack), sports betting, poker, scratch cards, bingo, slot machines outside of casino games (eg, in arcades or bars), and social gambling (eg, private gambling events with friends), tombola, and horse or other racing. The platform used for gambling was also registered (online vs offline). Answers were given on a 5-point Likert scale, ranging from 0 (“Never”) to 4 (“4 or more times a week”).
5. The Total Profit or Loss item was designed to capture monetary winnings or losses over the last 28 days. In Switzerland, profit or loss was captured in CHF (Swiss Franc), whereas Korea used KRW (Korean Won).
6. Two sleep metrics items were designed to assess participants’ sleep quantity and quality, asking participants about how many hours of sleep (0=“Less than 5 hours,” 5=“More than 9 h”) they usually get and how they would rate their overall sleep quality during the past 28 days (0=“Terrible,” 10=“Excellent”).
7. Two Level of Exercise items assessed participants’ physical activity over the last 28 days, asking them on how many days they were physically active for at least 15 minutes and how long they are usually physically active for (in min).
8. The Perceived Stress Scale (PSS-4) is a widely used brief 4-item instrument to assess stress [37,38]. It contains four items scored on a 0 (“Never”) to 4 (“Very often”) Likert-scale. Scores range from 0 to 16 (higher scores=higher perceived stress).
9. The Patient Health Questionnaire-9 is a reliable and validated 9-question tool developed to assess participants’ degree of depression. Answers are given on a 4-point Likert scale (0=“never,” 3=“almost always”) [39,40]. Scores range from 0 to 27 (0-4=minimal, 5-9=mild, 10-14=moderate, 15-19=moderately severe, 20-27=severe).
10. The Generalized Anxiety Disorder-7 Scale is a 7-item self-report questionnaire to estimate the severity of generalized anxiety disorder [41,42]. Its items ask about nervousness, inability to stop worrying, excessive worry, restlessness, difficulty relaxing, easy irritation, and the fear of something awful happening. Answers are given on a 4-point Likert scale (0=“never,” 3=“almost every day”). Scores range from 0 to 21 (0-4=minimal, 5-9=mild, 10-14=moderate, 15-21=severe).
11. The Alcohol Use Disorder Identification Test (AUDIT-C) is a 3-item tool screening for risky drinking and alcohol use disorders, focusing on the frequency of drinking, typical amount consumed, and heavy drinking episodes [43,44]. Scores range from 0 to 12 (higher scores=greater risk).
12. Consumption of Nicotine, Cannabis, and Other Drugs assessed single questions measuring the frequency of nicotine, cannabis, and other drug use over the past month. Responses range from 0 (“never”) to 4 (“daily or almost daily”). Scores were 0-4 for each substance (higher scores=more frequent use).
13. The Adult Self-Report Scale (ASRS) is a 6-item scale for attention-deficit/hyperactivity disorder (ADHD) screening, including questions about trouble completing tasks and remembering obligations [45,46]. Responses range from 0 (“never”) to 4 (“very often”) on a 5-point Likert scale. Scores were 0-24 (higher scores=more severe ADHD symptoms).
14. The P4 Suicide Screener is a brief 4-item measure to assess potential suicide risk [30]. If an elevated risk of suicide (scoring ≥ “minimal risk”) is recognized at any of the 3 assessments, the participant will not be eligible to participate in the study.
15. A self-developed questionnaire on study effects was designed to assess potential negative impacts of the study on participants’ well-being and behavior. Topics include gambling cravings, gambling behavior, and effects from wearing the smartwatch (eg, sleep quality, general well-being, and daily activities). Responses are measured on a 5-point Likert scale (0=“Not at all,” 4=“Very strongly”), with open-ended questions for additional feedback.

### Technical Specifications: Data Collection, Storage, and Security

EMA, baseline, and follow-up data were collected and stored using REDCap (Research Electronic Data Capture; Vanderbilt University) version 13.7.13 and MariaDB (MariaDB plc) version 10.11.13. All data were securely stored in Switzerland on servers of our institute’s hosting provider (NovaTrend Services GmbH) in accordance with current data protection regulations and using the latest security protocols, including encrypted data transmission (Transport Layer Security) and access controls. In Switzerland, a customized stand-alone app was developed for the 2nd generation Apple Watch SE using Xcode version 16.2 and Swift version 6.0.3 (6.0.3.1.10) to collect the physiological indicators that are relevant for this study (refer to “Measurement of Physiological Indicators” in the “Measurements” section for an overview and further details regarding the physiological indicators collected by the Apple Watch). The data collection process was designed to be entirely independent of a paired iPhone (Apple, Inc), ensuring that all indicators are collected and processed directly on the Apple Watch. Once collected by the app and synchronized by the participant, the data were transferred to Microsoft Azure Event Hubs, which accepted the incoming data for further processing. The further processed data was eventually stored in a MongoDB (MongoDB, Inc) database.

In Korea, data collection and processing were managed through a dedicated mobile app developed by the Andreia team. Participants installed this app on their phones and completed an in-app consent process to provide access to their health information. Following consent, the app collected physiological data—such as heart rate, heart rate variability, step count, and sleep patterns—from the Apple Watch by integrating with Apple’s HealthKit framework. These data were deidentified, linked to a unique participant identifier, and then securely transmitted to an AWS (Amazon Web Services) cloud server running Linux. The entire process was protected by security measures, including Transport Layer Security encryption for data transmission and AWS Security Group with IP-based access controls for the server, ensuring participant privacy in accordance with Apple’s data access protocols.

### Data Management and Analysis Plan

#### Overview

The primary outcome of interest will be whether participants report having gambled or not (ie, whether a gambling episode had occurred or not). Thus, the primary goal of our analysis will be to develop models that can reliably predict gambling behavior by using the information from the data that were obtained from the collection of the EMA and physiological smartwatch data. At present, data collection has been completed independently by both the Swiss and Korean research teams. The datasets containing the baseline, follow-up, EMA, and physiological smartwatch data from both countries will be merged to create a combined, cross-national dataset, which will serve as the basis for all analyses outlined in this protocol.

One prediction model we aim to develop will be based on explorative ML techniques, such as TSF or RNNs, to predict gambling episodes. ML models are capable of capturing complex, nonlinear relationships and interactions among multiple psychological and physiological indicators—patterns that may be missed by conventional (linear) approaches. Another advantage is the capability of these models to identify temporally dynamic patterns (eg, changes in craving levels or heart rate variability preceding gambling episodes), allowing us to explore whether short-term fluctuations or longer-term trends in internal states might predict an increased risk for gambling behavior.

Since ML models are sometimes hard to grasp with regard to the specific role of individual predictors (they are often considered “black boxes,” hardly seeing which predictor has a stronger effect compared to another), we will also use more classical (linear) models, such as GLMMs, which allow testing for statistical inference and interpretable coefficients and may provide additional insights into whether and how strong a certain predictor (eg, craving) is associated with the outcome (eg, gambling episode). In addition to GLMMs, mlVAR models have been discussed as a promising alternative to other more conventional methods (eg, linear approaches, such as GLMMs) when handling EMA data from addiction research studies [47]. A key advantage of mlVAR in the context of longitudinal data is its ability to explicitly model the temporal and reciprocal relationships between multiple variables across time. Unlike GLMMs, which primarily estimate how predictors at the same time point relate to an outcome in a unidirectional way, mlVAR allows us to examine how changes in psychological and physiological states (eg, craving and HRV) at one time point predict changes in gambling behavior at subsequent time points—and vice versa—providing a dynamic network of bidirectional effects. This enables a more nuanced understanding of dynamic processes and potential feedback loops underlying gambling episodes.

#### Data Preprocessing

After data collection, the data from both the EMA surveys and the physiological smartwatch sensors undergo several preprocessing steps to ensure data quality and consistency, enabling reliable analysis and modeling. Data preprocessing in this study includes handling missing values, noise filtering, data synchronization, normalization and scaling, feature engineering, and data augmentation.

1. Handling of missing values: Missing values are imputed using simple imputation (mean, median, and mode), time-series interpolation (eg, linear and spline), and ML–based techniques, such as K-Nearest Neighbors and Multiple Imputation by Chained Equations.
2. Noise filtering: Noise from physiological data is filtered using techniques, such as moving averages and Kalman filters.
3. Data synchronization: EMA and physiological data streams are synchronized along a time axis, with resampling applied as needed to create a consistent time series.
4. Normalization and scaling: All variables are adjusted using minimum-maximum scaling, standardization (*z*-scores), and robust scaling.
5. Feature engineering: New features are created from raw data, and the most predictive features are selected.
6. Data augmentation: The dataset is expanded using techniques such as Synthetic Minority Oversampling Technique and various time-series augmentation methods.

#### Exploratory Data Analysis

Exploratory data analysis (EDA) will be conducted to understand the characteristics of the collected data and identify key variables. EDA will help uncover associations between the data obtained from the EMA assessments (eg, internal states like craving intensity) and the physiological data from the smartwatch (eg, heart rate variability) and assess its significance. EMA and smartwatch data will be visualized to examine distributions, outliers, and missing data to develop a deeper understanding of the data, which in turn may be used for further prediction model development on the primary outcome variable.

#### Development of the ML Prediction Model

As the biosignal acquired from the smartwatch is basically time-series data, we plan to develop both a deep learning classification model using RNN or a 1D convolutional neural network model and shallow ML models specialized for time-series dataset, such as TSF to focus on interval information of the data, random interval spectral ensemble to use frequency information, and the shapelet classifier [48] to extract information from phase-independent similarity among biosignals. Those multiple ML classifiers will form a single voting ensemble model to maximize accuracy. The Python (Python Software Foundation) language will be used for EDA and model implementation, with common open-source libraries, including PyTorch, Pandas, and Keras. For model training and validation, a cloud-based server with an Nvidia V100 GPU will be used. The *F*_1_-score, the weighted average of precision and recall, will be used to measure the performance of the classification model, because both accuracy and specificity matter. It depends on the dataset and task, but normally, a classifier with an *F*_1_-score greater than 0.7~0.8 is reported to be good. This classifier can later be used in digital self-help program tasks as well, where participants wearing a smartwatch can receive interventions based on the biosignal containing information about their levels of feelings or cravings measured and uploaded in real time. With the uploaded biosignal, we can send off the participants’ necessary nudge interventions according to the level of the internal states of the participants accurately classified by the previously learned ML model.

#### GLMM Model

Given the hierarchical structure of repeated measurements nested within individuals, we will use GLMMs to analyze our longitudinal data and examine the associations between the collected indicators and gambling outcomes. We will develop models for our primary outcome of interest (ie, the occurrence of gambling episodes) and may develop additional models for secondary outcomes (eg, gambling craving intensity). Predictors will include both time-varying covariates reflecting the EMA data indicators (craving, stress, boredom, vitality, anxiety, and depression) and physiological indicators from the Apple Watch (HR, HRV, sleep metrics, and physical activity levels). The models will include random intercepts to account for individual differences in baseline gambling risk and random slopes to capture individual differences in predictor effects (provided the models achieve satisfactory convergence). Time-lagged versions of predictors will be included to examine temporal precedence. Analyses include both current (same-day effects) and prospective associations (next-day effects) between predictors and outcomes. Country (Switzerland vs Korea) will be included as a between-person factor to explore potential cultural differences in the outcome variables. Model selection will be based on a combination of information criteria (Akaike Information Criterion and Bayesian Information Criterion), theoretical considerations, and model interpretability. A model will be fitted using the *lme4* package (version 1.1.37; [49]) in R (version 4.4.1; R Foundation for Statistical Computing [50]).

#### mlVAR Model

We will use mlVAR models to examine the dynamic temporal relationships and feedback loops between the indicators collected via EMA (craving, stress, boredom, vitality, anxiety, and depression), physiological indicators collected by the Apple Watch (HR, HRV, sleep metrics, and physical activity levels), and gambling episodes. These models capture both contemporaneous and lagged associations in longitudinal data while accounting for individual differences in these temporal dynamics. The mlVAR approach will model the network of relationships between indicators collected by the EMAs, the physiological indicators collected by the Apple Watch, and gambling episodes. This approach allows us to identify which indicators predict changes in other variables over time (temporal networks), which variables co-occur within the same measurement occasion (contemporaneous networks), and how these relationships vary between participants (between-person networks). We will estimate separate mlVAR models for the Swiss and Korean samples to explore potential cultural differences in the dynamic interplay of predictors. Time-lagged effects will be examined using multiple intervals (t-1, t-2) to capture both immediate and delayed influences on gambling behavior. The analysis will reveal temporal patterns and reciprocal influences between indicators that can be used to develop just-in-time adaptive interventions (JITAIs) or EMIs, respectively. All mlVAR models will be fitted with the *mlVAR* package (version 0.5.2; [51]) in R (version 4.4.1; [50]).

### Sample Size

In the case of deep learning, approximately 5000 pieces of learning data per category are required to show acceptable performance [52]. Thus, the data size generally requires 1000-10,000 observations, and using 10,000 observations as a development dataset can detect a 0.1% performance improvement [53]. Therefore, the sample size of this study will be 100 participants (50 in Korea and 50 in Switzerland). If 100 participants are tracked for 28 days, 8400 records are generated (100 participants×28 d×3 EMAs per day), which is sufficient for ML modeling even when missing data is taken into account [54]. For multilevel models, studies suggest that for moderate effect sizes, a minimum of 50 participants (cluster level) with 20-30 observations provides adequate power (>0.80) to detect within-person effects [55,56].

### Ethical Considerations

The study has been approved by the Ethics Committee of the Faculty of Arts and Sciences at the University of Zürich (approval number 24.05.11; date of approval June 6, 2024) (Multimedia Appendix 1; Peer Review Report 1; Peer Review Report 2; Peer Review Report 3) and the Institutional Review Board (IRB) of Uijeongbu St. Mary’s Hospital, Catholic University of Korea (IRB number UIRB-정20240725‐022; date of approval July 26, 2024). The study will be executed in compliance with the Helsinki Declaration. All participants will be provided with an informed consent form and given the opportunity to decide whether or not to participate. The informed consent form provides detailed information about the purpose, procedures, potential risks, compensation, and privacy protections associated with the study. All data will be anonymized to ensure that individual subjects cannot be identified. The data collected in this study will be used for research purposes only. Access to the data will be limited to the principal investigators and authorized researchers.

## Results

At the time of the protocol submission, recruitment and data collection have been completed by both the Swiss and Korean research teams (IRRID: DERR1). In Switzerland, a total number of 52 Swiss participants (mean age 37.4, SD 11.9; range 18‐79 years; female: n=5, 9.6%) were recruited between February and April 2025. In Korea, a total number of 57 participants (mean age 25.6, SD 4.5, range 19‐33 years; female: n=29, 50.9%) were recruited between October and December 2024. The datasets of both countries are currently being merged and prepared for all further statistical analyses (eg, the development of prediction models), which will be performed as outlined in the “Methods” section once the data merging is complete and a combined cross-national dataset is created. Hypothesis testing has not been conducted yet and will be conducted once the dataset is merged and finalized. Results will be published in a scientific peer-reviewed journal. At present, only descriptive statistics (eg, sample characteristics) have been analyzed for each national sample and have been used to present preliminary results at conferences. The Korean team has previously tested their ML model for internal purposes using the dataset from the Korean participants; the Swiss sample was not included. Anonymized study data will be available on request. Participants will be informed via email about study results via a layperson-friendly summary of trial findings if they have requested so at registration.

## Discussion

### Principal Findings

Building on the findings of the first EMA and EMI studies in the field of gambling [23,24], this study will be the first to examine the influence of potentially relevant physiological indicators that can be collected continuously and nonintrusively using wearable devices (ie, an Apple Watch). In addition to the wealth of EMA and physiological wearable data (BioMeTs) collected, the planned study is innovative in its use of ML methodology to develop prediction models. Unlike traditional regression models, which rely heavily on linear relationships between variables, this study uses advanced ML methodologies, such as RNNs and TSFs, to analyze complex, nonlinear interactions between physiological and psychological indicators. This approach is expected to yield predictive models with higher accuracy, thus contributing to the growing field of digital health interventions for behavioral addictions. Since the task involves building an accurate classification model for use in early intervention (eg, EMIs), we opted for ML methods.

As clients rarely report addiction problems accurately, determining the timing and intensity of appropriate intervention is difficult. Therefore, if a model based on EMA and/or physiological wearable device data could be created that predicts gambling behavior with high accuracy, this would be a valuable technique for enabling early interventions with clients who have (behavioral) addiction problems, paving the way for JITAIs, such as EMIs. Additionally, developing physiological data analysis that can predict behavioral addiction through ML techniques would provide important evidence for the development of an integrated biopsychosocial behavioral addiction model. The use of wearable devices also addresses a significant limitation in addiction research: reliance on self-reported data. Wearables provide a less intrusive, more reliable alternative by passively collecting continuous physiological data. This capability enables more precise and timely detection of changes in participants’ stress levels [57,58], sleep patterns [59,60], and physical activity, all of which are closely linked to gambling behavior.

### Expected Results

A significant advantage of this research lies in its potential for providing early interventions just in time for individuals who need it the most. It is expected that variables collected via EMAs and wearable devices (in our case, the Apple Watch) will enable the development of valid prediction models for gambling episodes and cravings. The incorporation of the relevant variables in existing digital self-help interventions that address problem gambling and the provision of just-in-time, individually tailored intervention elements could improve program engagement and effectiveness. This allows for objective prediction before a gambling episode becomes “serious,” allowing for effective gambling episode prevention. It is expected that stress in particular, measured by HR and its variability, physical activity, and both sleep duration and quality could be good predictors of gambling cravings and gambling episodes [57-60]. We assume that these physiological indicators have a higher predictive value than the corresponding self-reported constructs measured via EMAs because the device continuously, passively, and nonintrusively measures and collects indicators throughout the day, without individuals having to report their current state actively (eg, stress levels). This goes along with the assumption that fluctuations in certain states can be more easily detected compared to self-reported EMA surveys, which usually require individuals to complete short questionnaires several times throughout the day to report their current states.

### Challenges and Future Directions

Despite the expectation of promising results, the study may also face several challenges. It is essential to implement robust engagement strategies to ensure that participants comply with the instructions to wear the Apple Watch for extended periods of time and complete the EMA surveys. To mitigate the risk of low participant engagement, we implemented several measures, such as a monitoring strategy, reminders, and proactive follow-ups, as well as an incentive plan.

Moreover, the efficacy of the ML models is contingent upon the quality and consistency of the data collected. The presence of missing or erroneous data could potentially compromise the performance of the model, necessitating the implementation of advanced preprocessing techniques.

Furthermore, as a cross-national study between Switzerland and Korea, this research faces challenges arising from methodological differences. For instance, the data synchronization method and the compensation scheme varied between the 2 countries. In Switzerland, participants had to manually synchronize their data once a day, whereas in Korea, data were synchronized automatically when participants accessed the app for EMA surveys. Additionally, the Swiss cohort received a graded reward based on their level of engagement, while the Korean cohort was subject to strict dropout criteria, receiving a uniform reward only upon maintaining high compliance. These different incentive structures and synchronization methods may have uniquely influenced participant motivation and data collection patterns in each country. Acknowledging these differences is crucial for interpreting the study’s findings.

Future research could investigate the integration of supplementary data sources, such as geolocation or social media activity, with the aim of enhancing the predictive accuracy of the model.

### Broader Impact

The insights gained from this study have implications beyond gambling addiction. The methodologies and findings could be applied to other behavioral and substance-related addictions, thereby enhancing the scope of digital health interventions. Wearable technology is already widespread and offers a scalable solution to public health challenges by enabling remote monitoring and intervention. Consequently, this study aligns with global efforts to leverage technology to improve mental health outcomes and reduce health disparities. Identifying good physiological indicators for predicting mental health or addictive behaviors, such as depressive episodes, nicotine use, or alcohol consumption, could take the personalization, adherence, and effectiveness of digital interventions to the next level. In particular, populations that encounter obstacles when accessing conventional mental health services, such as those living in remote regions or individuals who are stigmatized, could benefit greatly from wearable-enabled interventions. The anonymized, low-threshold nature of these interventions enhances their acceptability and potential reach.

### Conclusion

This longitudinal, cross-national observational study combines EMAs with continuously collected physiological data from Apple Watches to identify predictors of gambling episodes and gambling craving in at-risk gamblers. Self-reported states (eg, levels of craving, stress, boredom, vitality, anxiety, and depression) will be combined with passively collected biosignals (eg, heart rate, heart rate variability, sleep metrics, and activity indicators). The goal is to describe time-varying risk patterns that precede gambling. Using two complementary analytic approaches—machine learning for high-dimensional prediction and multilevel modeling for interpretable within-person and between-person effects—we will develop and compare prediction models for short-term gambling risk. The findings are expected to enable the development of just-in-time adaptive ecological momentary interventions that provide targeted support when risk is elevated (ie, when participants are in need of support) while keeping user burden low. The cross-national design also allows for examining potential cultural differences in predictors and model performance. Overall, this protocol provides a scalable framework for using wearable and EMA data to improve digital interventions for gambling and other addictive behaviors.

## Supplementary material

10.2196/82782 Multimedia Appendix 1 Decision letter from the funding agency.

10.2196/82782 Peer Review Report 1 Peer-review report nr. 1 from a granting agency.

10.2196/82782 Peer Review Report 2 Peer-review report nr. 2 from granting agency.

10.2196/82782 Peer Review Report 3 Peer-review report nr. 3 from a granting agency.

## Abbreviations

ADHD: attention-deficit/hyperactivity disorder
ASRS: Adult Self-Report Scale
AUDIT-C: Alcohol Use Disorder Identification Test
AWS: Amazon Web Services
BioMeT: biometric monitoring technology
*DSM-5*: *Diagnostic and Statistical Manual of Mental Disorders* (Fifth Edition)
EDA: exploratory data analysis
EMA: ecological momentary assessment
EMI: ecological momentary intervention
G-SAS: Gambling Symptom Assessment Scale
GLMM: generalized linear mixed-effects model
HR: heart rate
HRV: heart rate variability
*ICD-11*: *International Classification of Diseases 11th Revision*
IRB: Institutional Review Board
JITAI: just-in-time adaptive intervention
ML: machine learning
mlVAR: multilevel vector autoregression
PGSI: Problem Gambling Severity Index
PSS-4: Perceived Stress Scale
REDCap: Research Electronic Data Capture
RNN: recurrent neural network
TSF: time series forecasting
