# Supplementary material for: Identifying Predictors of Gambling Episodes and Craving Using Ecological Momentary Assessment and Smartwatch-Based Physiological Measures: Protocol for a Longitudinal Observational Study
Source: JMIR Res Protoc. 2026 Feb 19;15:e82782. doi: 10.2196/82782 (PMC12919748; doi:10.2196/82782)
Supplement: Peer Review Report 1 [file resprot-v15-e82782-s002.pdf]

Review: 1

Application data

Applicant(s)
Schaub, Michael

Leveraging the potential of digital interventions for addictive behaviors by ecological momentary interventions (EMI) including biometric monitoring technologies (BioMeTs)
Korean-Swiss Science and Technology Programme (KSSTP)

Detailed evaluation

Scientific relevance

Specific strengths

This project could potentially identify some physiological characteristics that indicate the likelihood that people addicted to gambling are at risk of engaging in harmful behaviour. This could in turn provide some insight into how they might be provided with brief interventions to assist in avoiding this harmful behaviour. This could certainly provide support to people participating in the intervention. Identifying the physiological markers of a propensity to engage in harmful gambling may be a useful step towards providing support and interventions to prevent such harmful behaviour.

Specific weaknesses

There are major ethical issues with the collection and analysis of data relating to the physiological markers of gambling involvement. The data collection and analysis systems utilised in the trial, and in particular any widespread implementation, need to be very carefully protected, to prevent any exploitation (inadvertent or otherwise) of the data and to ensure that the privacy and interests of those participating are protected to the greatest possible degree.
The language used in the application to describe those experiencing gambling disorder ('problem gamblers') is archaic and associated with the gambling industry discourse of 'responsible gambling'. This is very unhelpful for people struggling with gambling disorder and needs to be corrected.
The intervention may be useful for some people, but evidence of the uptake of other interventions likely to provide support to those experiencing gambling disorder suggests that only a small proportion of those who might benefit actually utilise the opportunity to participate - e.g., counselling, self-exclusion, voluntary pre-commitment, etc. There is no reason to suggest that the proposed intervention would be any different, and there is no reason to suggest that continued involvement in the intervention could be guaranteed over any length of time.
The proposed intervention is not preventive, and only marginally/moderately likely to minimise harm. It relies on compliance and on individuals remembering to wear a device during periods when they might be stressed or anxious.

Comments

Although this proposal may assist in identifying physiological characteristics associated with gambling harms, I am not convinced that it would lead to an effective intervention. It involves active and continued cooperation on the part of people experiencing gambling disorder, which can be a very unsettling and difficult disorder. It also involves significant ethical risks, involving personal data that, if subject to data hacking or leaking, could be exploited to the great detriment of participants. Systems to manage these risks must be very carefully designed and implemented.
There is solid evidence that people experiencing gambling disorder are unlikely to take advantage of support or interventions that might assist them - such as self-exclusion, counselling, voluntary pre-

commitment education or other supportive measures. Further, it relies on individuals remembering or continually choosing to wear a device. This may be unlikely for reasons referred to above.

Originality of aims and objectives

Specific strengths

The aims and objectives are of moderate originality.

Specific weaknesses

The principal weakness of this project is its reliance on downstream interventions that may have limited efficacy in practice.

Comments

Please see comments under scientific relevance.

Appropriateness of the methodology and feasibility

Specific strengths

The funds involved are significant and may lead to recruitment of the necessary number of research participants.

Specific weaknesses

The continued and continuous utilisation of the wearable device by research participants may be uncertain.

Comments

See above.

Track record and expertise of the researchers

Specific strengths

The researchers have excellent track records and appear well capable of undertaking the project.

Specific weaknesses

There are no specific weaknesses in the backgrounds or scientific track records of the CIs

Comments

Complementarity of the research partners

Specific strengths

The research partners appear to be well integrated.

Specific weaknesses

There are no specific weaknesses identified.

Comments

Comment

I have no concern with the track records of the CIs, and their scientific output is impressive. However, the proposal is of likely modest benefit to the field, and I am not convinced that it would lead to an effective intervention to address gambling disorder. I am much more inclined to think that if implemented it might provide marginal/moderate benefits to some people - but certainly not to the majority of those who experience gambling disorder. At this stage in the evolution of the field of gambling addiction it is likely that preventive measures are much more likely to be efficacious, and this is at best a harm minimisation intervention, with likely limited uptake from those for whom it might provide some benefits. I believe that the effects of this intervention, if developed and implemented, would most likely be of marginal or at best moderate utility for the prevention of gambling harm. I am unaware of the total resources available to this funding source, but the commitment of hundreds of thousands of Francs to this project may involve opportunity costs to other research focused on possible more broadly beneficial goals.

Note on the evaluation procedure

The evaluation bodies of the SNSF strive to reach a balanced overall assessment of each proposal. External reviews play an important role in this. Reviewers generally review only one proposal. The evaluation bodies of the SNSF, however, must compare and rate the quality of all proposals submitted by a given deadline. The opinions expressed in external reviews are generally positive, or they may occasionally include critical remarks that are largely irrelevant to the assessment conducted by the evaluation body. Therefore, the final decision taken by the SNSF evaluation bodies need not necessarily reflect the content of external reviews.
