# Supplementary material for: Identifying Predictors of Gambling Episodes and Craving Using Ecological Momentary Assessment and Smartwatch-Based Physiological Measures: Protocol for a Longitudinal Observational Study
Source: JMIR Res Protoc. 2026 Feb 19;15:e82782. doi: 10.2196/82782 (PMC12919748; doi:10.2196/82782)
Supplement: Peer Review Report 2 [file resprot-v15-e82782-s003.pdf]

Review: 2

Application data

Applicant(s)
Schaub, Michael

Leveraging the potential of digital interventions for addictive behaviors by ecological momentary interventions (EMI) including biometric monitoring technologies (BioMeTs)
Korean-Swiss Science and Technology Programme (KSSTP)

Detailed evaluation

Scientific relevance

Specific strengths

- The topic is a timely one, given the increase in online gambling worldwide and the ongoing debates around the conflation between gambling and gaming through devices such as loot boxes.
- The ECM approach is interesting and potentially highly valuable for intervening with problem gambling, especially problem gambling where interventions can be sent in real time.
- The planned pilot study in Korea include elements of co-creation, which is increasingly seen as a vital component in meaningful gambling harms research.

Specific weaknesses

- The dissemination section does not include any information on dissemination plans. Given the societal impact of gambling it would have been good to see some discussion around engagement with stakeholders, including those from beyond academia. In other words, ensuring that the scientific relevance is recognised by those in a position to implement the results of the research in practical terms.

Comments

Originality of aims and objectives

Specific strengths

The aims and objectives are novel. I am not aware of any research that done what the authors are proposing here.

Specific weaknesses

As noted with reference to the methodology, I would question where people recruited from gambling treatment centres really meet the criteria of being 'general population'

Comments

Appropriateness of the methodology and feasibility

Specific strengths

- Aside from the potential issues with recruitment, the overall methodology appears to be feasible. The study is in itself a feasibility study, and so I feel that valuable information can still be obtained from it even if it does not fully meet all of the planned objectives.

Specific weaknesses

- The main limitation in the research design is that it will consist of a single group being tested over a period of time in a longitudinal study. It could be useful to consider including a second group of people who are gamblers, but who are not problem gamblers (i.e. PGSI < 8), although I appreciate that it is often harder to recruit non-problem gamblers from the general population than it is to recruit problem gamblers.
- Linked to the above, I would query if there could be issues recruiting the sample from a mixture of online sources and those already in contact with gambling counselling services. These could potentially be two quite different populations. I would recommend exploring whether the full sample can be collected from one of these sources or, if not, attempting to balance the numbers out between sources.
- From our own experience I would caution that there could be an issue of study drop out with population. I appreciate that adherence rate is one of the things the authors will be looking at, but of course low adherence is different from completely withdrawing from the study, or just ceasing contact with the researchers. It would good to consider if the planned sample size will allow for analysis to still go ahead as planned if drop out does occur.
- It is stated that the Korean Win Back Control pilot test will include people with a PGSI > 3. Maybe I am just missing something, but this seems like it a disconnect between this and the first study that will include people who have a PGSI of 8 or more.
- It is unclear why there is an age cut off of 50.
- It is not stated if there will be any attempt to reach a gender balance in the sample. This is something that it is important to be aware of. Given that many gambling treatment services are aimed primarily at men it can be very difficult to recruit female gamblers for research.

Comments

Track record and expertise of the researchers

Specific strengths

- The researchers both have experience in researching gambling, which is a benefit given how complex and challenging research into gambling can be.
- Both researchers also appear to be experienced in research and grant management in general.

Specific weaknesses

- None identified.

Comments

Complementarity of the research partners

Specific strengths

- The named research partner appears to have complimentary skills to the research team, particularly in relation to the setup of the technology required for the EMA and EMI.

Specific weaknesses

- None identified.

Comments

Comment

I believe that this is a novel and potentially impactful project into an important and topical societal issue. The research team appear to be fully qualified to conduct the research. My only real concerns are around some of the methodological aspects, and how easy these will be to achieve given the challenging nature of the topic and the realities of working with participants who are experiencing gambling harms.

Note on the evaluation procedure

The evaluation bodies of the SNSF strive to reach a balanced overall assessment of each proposal. External reviews play an important role in this. Reviewers generally review only one proposal. The evaluation bodies of the SNSF, however, must compare and rate the quality of all proposals submitted by a given deadline. The opinions expressed in external reviews are generally positive, or they may occasionally include critical remarks that are largely irrelevant to the assessment conducted by the evaluation body. Therefore, the final decision taken by the SNSF evaluation bodies need not necessarily reflect the content of external reviews.
