# Supplementary material for: Identifying Predictors of Gambling Episodes and Craving Using Ecological Momentary Assessment and Smartwatch-Based Physiological Measures: Protocol for a Longitudinal Observational Study
Source: JMIR Res Protoc. 2026 Feb 19;15:e82782. doi: 10.2196/82782 (PMC12919748; doi:10.2196/82782)
Supplement: Peer Review Report 3 [file resprot-v15-e82782-s004.pdf]

Review: 3

Application data

Applicant(s)
Schaub, Michael

Leveraging the potential of digital interventions for addictive behaviors by ecological momentary interventions (EMI) including biometric monitoring technologies (BioMeTs)
Korean-Swiss Science and Technology Programme (KSSTP)

Detailed evaluation

Scientific relevance

Specific strengths

This project is well considered and will likely make a useful and novel contribution to the scientific literature on the problematic gambling as well as contributing to advances in policies and practices to reduce gambling harms

Specific weaknesses

It would be useful for the applicants to expand further on their own work on EMIs

Comments

Originality of aims and objectives

Specific strengths

There has been limited research on ecological momentary interventions for gambling. These have a sound rationale as gambling is associated with experience of distress and negative emotions as well as being situational dependent and triggered by cues. As such, the ability to overcome the urge to gamble in the moment and identify and respond proactively and positively to triggers may be helpful in harm reduction. The topic is novel and significant.

Specific weaknesses

it would be useful to have more detail on the study contribution to theoretical approaches to gambling harm prevention

Comments

Appropriateness of the methodology and feasibility

Specific strengths

The methodology is appropriate to address the aims. A significant advantage of the study is the cross-cultural aspect which will examine the feasibility an effectiveness of EMI-based interventions across very different cultural groups. This overcomes a chief limitation of many studies in terms of geographic and cultural isolation.

Specific weaknesses

- What is the rationale for exclusion of participants >50 years of age for the initial study?
- Will there be an effort to ensure even distribution of gender in studies?
- What is the anticipated sample size based on? Have power calculations been conducted?
- It is noted that previous interventions using EMI have issues with compliance and adherence. How will this be managed in this program including trying to enhance compliance and managing lack of compliance?

Comments

Track record and expertise of the researchers

Specific strengths

The research team are appropriately experienced and they have complementary expertise.

Specific weaknesses

nil

Comments

Complementarity of the research partners

Specific strengths

The research team are appropriately experienced and they have complementary expertise.

Specific weaknesses

More details on project management would be helpful

Comments

Comment

sound project with innovative potential contribution to field and practical outcomes

Note on the evaluation procedure

The evaluation bodies of the SNSF strive to reach a balanced overall assessment of each proposal. External reviews play an important role in this. Reviewers generally review only one proposal. The evaluation bodies of the SNSF, however, must compare and rate the quality of all proposals submitted by a given deadline. The opinions expressed in external reviews are generally positive, or they may occasionally include critical remarks that are largely irrelevant to the assessment conducted by the evaluation body. Therefore, the final decision taken by the SNSF evaluation bodies need not necessarily reflect the content of external reviews.
