# Supplementary material for: Identifying Predictors of Gambling Episodes and Craving Using Ecological Momentary Assessment and Smartwatch-Based Physiological Measures: Protocol for a Longitudinal Observational Study
Source: JMIR Res Protoc. 2026 Feb 19;15:e82782. doi: 10.2196/82782 (PMC12919748; doi:10.2196/82782)
Supplement: Multimedia Appendix 1 [file resprot-v15-e82782-s001.pdf]

Schweizerischer Nationalfonds

Internationale Zusammenarbeit
Wildhainweg 3, Postfach
3001 Bern

+41 31 308 22 22
international@snf.ch
www.snf.ch

Herr Prof. Michael Schaub
Schweizer Institut für Sucht- und
Gesundheitsforschung ISGF
Konradstrasse 32
Postfach
CH-8005 Zürich

Bern, 22. September 2023

Verfügung
Beitrag Nr. IZKSZ1_218534 / 1

Sehr geehrter Herr Prof. Schaub

Der Schweizerische Nationalfonds (SNF) und die *National Research Foundation* (NRF) in Korea haben Ihr Gesuch mit dem Titel «Leveraging the potential of digital interventions for addictive behaviors by ecological momentary interventions (EMI) including biometric monitoring technologies (BioMeTs)» gemeinsam evaluiert. Wir freuen uns, Ihnen mitzuteilen, dass beschlossen wurde, Ihr Projekt zu unterstützen. Der SNF bewilligt dafür einen Forschungsbeitrag von **CHF 249'958**.

Per 14. Februar 2023 sind 37 Forschungsgesuche eingegangen, welche sowohl beim SNF als auch bei der NRF antragsberechtigt waren. Insgesamt können 12 Projekte mit einem Gesamtbetrag von CHF 3 Mio. gefördert werden, was einer Bewilligungsquote von 32 Prozent entspricht. Es können nur die Gesuche des ersten Quintils sowie gewisse Gesuche des zweiten Quintils finanziert werden.

Der SNF spricht in der Regel Globalbudgets mit festgelegten Jahrestranchen zu. Sie sind im Rahmen der reglementarischen Bestimmungen und unter Berücksichtigung der in diesem Schreiben genannten Bedingungen frei, wie Sie die Mittel einer Jahrestranche auf die anrechenbaren Kosten verteilen. *my*SNF bietet Ihnen Unterstützung bei der Verwaltung Ihres Beitrages. Sie können Ihr Budget online einsehen und bearbeiten.

Zugesprochene Jahrestranchen in CHF

Total	1. Tranche	2. Tranche	3. Tranche
249'958	93'926	80'201	75'831

Beginn: 1. Oktober 2023 Dauer: 36 Monate

Im Falle von Kürzungen der Bundesmittel, die das Budget des SNF tangieren, können Reduktionen bei den Tranchen nicht ausgeschlossen werden.

SNF

Verantwortliche/r Gesuchsteller/in Ausland

- Prof. Hae Kook Lee, Industry-Academic Cooperation Foundation Catholic University of Korea, Seoul

Projektpartnerinnen und Projektpartner

- Prof. Tobias Kowatsch, Institut für Implementation Science in Health Care, Medizinische Fakultät, Universität Zürich, 8006 Zürich

Geltende Rechtsgrundlagen

Die Rechtsgrundlagen für den vorliegenden Entscheid und den Beitrag sind das Ausschreibungsdokument, das Reglement über die Projektförderung (www.snf.ch/reglemente-pf), das Beitragsreglement (www.snf.ch/br) und das Allgemeine Ausführungsreglement zum Beitragsreglement (www.snf.ch/aar).

Informieren Sie allfällige weitere in das Vorhaben involvierte Personen und beachten Sie auch Ihre Informationspflicht gegenüber dem SNF.

Weitere wichtige Informationen finden Sie im Anhang. Für die Realisierung Ihres Vorhabens wünschen wir Ihnen viel Erfolg.

Freundliche Grüsse

Dr Delphine Marchon

Zusätzliche Informationen zu Ihrem Gesuch und zum weiteren Vorgehen auf *my*SNF

Weitere Angaben zur Beurteilung Ihres Gesuches finden Sie direkt in *my*SNF. Sie können unter «Dokumente > Anonymisierte Gutachten» auch anonymisierte externe Gutachten einsehen (frühestens zwei Tage nach dem Datum dieser Verfügung).
Zudem wird Sie *my*SNF auch bei den weiteren Schritten zur Beitragsfreigabe und -abwicklung unterstützen. Im Leitfaden für das Lifetime-Management von Forschungsgesuchen (Beiträgen) (www.snf.ch/lifetime) finden Sie dazu umfassende Informationen.

Open Access
Wir weisen darauf hin, dass Sie die Verantwortung dafür tragen, die Open-Access-Verpflichtung des SNF zu erfüllen. Diese gilt für alle wissenschaftlichen Publikationen, die durch die Förderung dieses Beitrags entstehen. Sie müssen unmittelbar oder innerhalb der zulässigen Embargofristen, unentgeltlich und uneingeschränkt, auf digitalem Weg verfügbar gemacht werden. Siehe dazu die Open-Access-Webseite des SNF: http://oa100.snf.ch

Open Research Data
Der SNSF erwartet, dass in den geförderten Projekten erhobene Daten in wissenschaftlich anerkannten Datenrepositorien, welche die FAIR Prinzipien erfüllen, öffentlich zugänglich sind, sofern keine rechtlichen, ethischen, urheberrechtlichen oder sonstige Probleme bestehen. Für bewilligte Beiträge wird ein Datenmanagementplan (DMP) für die Beitragsfreigabe verlangt. Siehe dazu die Open Research Data-Webseite des SNF: Open Research Data.
